# Effects of Acute Mental Stress on Choroidal Thickness

**DOI:** 10.3390/bioengineering11070684

**Published:** 2024-07-05

**Authors:** Jiechun Lin, Yingxiang Han, Meng Liu, Xiaofei Wang

**Affiliations:** 1 School of Ophthalmology and Optometry, School of Biomedical Engineering, Wenzhou Medical University, Wenzhou 325035, China; linjiechun98@163.com (J.L.); lm18391863349@163.com (M.L.); 2 Key Laboratory for Biomechanics and Mechanobiology of Ministry of Education, Beijing Advanced Innovation Center for Biomedical Engineering, School of Biological Science and Medical Engineering, Beihang University, Beijing 100083, China; hanyx1020@yeah.net

**Keywords:** mental stress, choroidal thickness, myopia, optical coherence tomography, autonomic nervous system

## Abstract

**Purpose:** Previous studies have indicated an association between education and myopia, suggesting that numerous stress events during the educational process may influence eye health. This study aimed to investigate the impact of mental stress induced by mental arithmetic (MA) on choroidal thickness (CT). **Methods:** This study included 33 participants aged between 19 and 29 years. Swept-source optical coherence tomography (SS-OCT) was used to capture images of the posterior segment of the left eye during baseline and MA to assess changes in the CT. After denoising and compensation, the baseline images and MA images that had been rigidly registered and resampled to the baseline images were segmented using a deep learning-based method. Based on the segmentation results, the CT within the regions of 1 mm and 3 mm diameter centered at the lowest point of the fovea was calculated. **Results:** Significant increases were observed in both CT_1mm_ and CT_3mm_ during MA, with mean changes of 2.742 ± 7.098 μm (*p* = 0.034) and 3.326 ± 6.143 μm (*p* < 0.001), respectively. **Conclusions:** Thickening of the choroid has been observed during acute mental stress. We speculate that long-term or chronic mental stress could have a potential adverse impact on myopia progression.

## 1. Introduction

Myopia, characterized as a refractive anomaly where light rays entering the eye parallel to the optic axis converge in front of the retina when accommodation is at rest, stands as a prevalent ocular disorder globally. There is a global increase in myopia prevalence. The highest rates were observed in East and Southeast Asia, where 80–90% of individuals were affected by the age of 18. This rate significantly exceeded those of Central Europe and Central Asia. It is projected that by 2050, approximately half of the global population will be affected by myopia, with around 10% suffering from high myopia [1,2]. The correction of myopia typically involves optical interventions such as eyeglasses, contact lenses, or refractive surgery. These methods adjust the focal point of light entering the eye to align with the retina, thereby improving visual clarity. Despite the effectiveness of these corrective measures, they do not address the underlying structural changes of the eye. 

Myopia can cause serious complications. These include myopic macular degeneration, retinal detachment, cataracts, and open-angle glaucoma, all of which can be serious and may even result in blindness [3]. Myopia has been listed as one of the five eye conditions that are an immediate priority by the World Health Organization’s Global Initiative for the Elimination of Avoidable Blindness [2]. While myopia can be somewhat managed through optical methods such as orthokeratology lenses, pharmacological treatments like atropine eye drops, and behavioral modifications, including increased outdoor activities, the precise mechanism of myopia is still a mystery [4,5]. The prevalence of myopia has significantly increased compared to the past two or three generations. However, the gene pool has not changed sufficiently to explain the rate of increase in myopia incidence [6]. This suggests that the prevalence of myopia, particularly among children, is more likely driven by behavioral and environmental changes rather than changes in genetic factors [7].

Classical epidemiological studies have identified a causal relationship between education and myopia. This relationship has been validated by Mendelian randomization, although the mechanisms involved remain unclear [6,8]. It is widely acknowledged that the educational environment is filled with stressful events, such as difficult assignments, intense examinations, time management, and class presentations. Events appraised as stressful by individuals or interpreted by the brain as real or potential threats are termed stressors, leading to the release of stress mediators. These mediator molecules interact with their corresponding receptors located in the periphery and the brain, resulting in the stress response [9,10,11]. The stress response is complex, involving multiple systems throughout the body, including the autonomic nervous system. Moreover, if an individual is exposed to a stressor, the autonomic nervous system is triggered: generally, the sympathetic branch is activated and the parasympathetic branch is inhibited when it involves mental work, active coping (i.e., taking action or exerting efforts to remove or circumvent the stressor), or the defense reaction [12,13,14,15].

A multitude of ocular structures receive innervation from the autonomic nervous system, such as the dilator pupillae muscle, the sphincter pupillae muscle, the ciliary muscle, the choroidal non-vascular smooth muscle, and the ophthalmic artery. An increase in sympathetic input or a decrease in parasympathetic input leads to vasoconstriction of the ophthalmic artery and its branches in the optic nerve, iris, ciliary body, choroid, and episclera and changes in the choroidal non-vascular smooth muscle tone [16]. Changes in the choroidal blood flow and non-vascular smooth muscle tone are suspected to affect the thickness of the choroid [17]. Additionally, the choroidal thickness (CT) has been proposed as a biomarker for the prediction of future axial elongation, with decreased axial elongation being related to a thicker choroid despite the existence of conflicting reports [18]. 

Mental stressors, which may affect the eyes through the autonomic nervous system, are common and unavoidable in educational environments. However, the effects of acute mental stress on the posterior segment of the eye have not been explored. Investigation into the choroidal response to mental stress may provide insights into the mechanisms of myopia. This study aimed to investigate the effects of acute mental stress induced by mental arithmetic (MA) [19] on the CT, using optical coherence tomography (OCT). As the heart is also regulated by the autonomic nervous system [20], heart rate (HR) data were collected during the experiment to assist in the assessment of stress status [21,22].

## 2. Methods

### 2.1. Subjects

This study included thirty-three healthy subjects from university students in the Beijing region of China. Participants with neurological disorders, systemic illnesses, organic eye diseases, or fixation difficulties were excluded from the study. The subjects were requested to avoid the consumption of coffee or alcohol for a 12 h period before the experiment. This precaution was taken to protect the autonomic nervous system from potential effects. Their axial lengths were measured by the IOLMaster 700 (Carl Zeiss Meditec AG, Jena, Germany). This study was approved by the Biological and Medical Ethics Committee of Beihang University and adhered to the principles of the Declaration of Helsinki. Informed consent was obtained from all study participants.

### 2.2. OCT Imaging and Heart Rate Measurements

Prior to the experiment, participants were asked to view a minimum of ten minutes of documentary clips. The clips were displayed on an 11-inch screen with a resolution of 2388 × 1668 pixels and a pixel density of 264 ppi, viewed from approximately 1 m away. The selected clips came from the ‘Mountains’ and ‘Great Plains’ episodes of *Planet Earth*, which focus on wildlife and nature. The clips were presented in a resolution of 720p and a frame rate of 25 frames per second. The selection was based on criteria of low cognitive demand, minimal arousal, emotional neutrality, and non-tediousness. Mandarin dubbing and Chinese subtitles are featured in all the clips. After viewing, participants were instructed to fixate on a distant object for at least ten seconds prior to the experiment to reduce the effects of accommodation [23].

**Figure 1:**
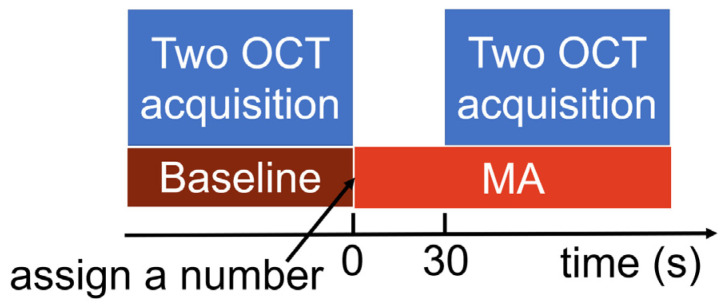
Experimental procedure.

The VG200D swept-source OCT device (SVision Imaging, Ltd., Luoyang, China), equipped with a laser of approximately 1050 nm in central wavelength, was used to obtain images of the posterior segments of the left eye before (i.e., baseline) and during the MA task. Under the MA condition, participants were instructed to silently subtract 17 serially from a three-digit number, randomly assigned and exceeding 900. They were emphasized to perform the calculations as quickly as possible. Participants were required to report the final answer after completion of all the imaging. Although the final answer was not used for analysis, this requirement could help to decrease the possibility of subjects relaxing. A raster scan protocol of 512 × 512 A-scans was used to acquire image data, covering a 12 mm × 12 mm area. Each scan consisted of an average of four frames, with a depth of 4.5 mm (1920 pixels). Eye movement tracking was turned on during OCT imaging to mitigate the effects of eye motion artifacts, thereby improving the clarity and reliability of the images obtained. Images of the posterior segment were captured twice at baseline, followed by two consecutive captures after 30 s of the MA, with the MA still ongoing. During imaging, participants were instructed to direct their gaze at the central internal fixation target. Additionally, participants were advised to close their eyes between each acquisition, including image saving, to alleviate eye strain and dryness. Generally, each image acquisition took between 25 and 40 s, excluding the time for adjustment and focusing. The experimental procedure is illustrated in Figure 1. Artificial tears were applied depending on whether participants had experienced symptoms of dry eye recently. A single examiner (JL) conducted all the examinations. 

The experiment was conducted between 14:00 and 22:00. Participants were clearly informed about the procedure before the experiment to minimize the interval between baseline and MA conditions. Throughout the experiment, the RR intervals—which represent the time between two consecutive R waves of the QRS signal on an electrocardiogram—were acquired. These data were captured at a frequency of 1000 Hz by the Polar H10 chest strap sensor (Polar Electro Oy, Kempele, Finland), and the Elite HRV application version 5.5.9 (Elite HRV Inc., Asheville, NC, USA) was used for data recording and storage.

### 2.3. OCT Image Processing and Segmentation

Typically, the analysis of the posterior segment structural parameters was conducted using the second volume (3D images) obtained during the baseline condition and the first volume obtained during the MA task. If the image displayed issues, including poor quality or being incomplete, it would be substituted with another image captured under the same conditions for the analysis. Every volume that was ultimately selected for the analysis possessed a signal strength of at least 7 out of 10. The transverse scale of the OCT images was adjusted according to individual axial length, based on Bennett’s formula, magnification ratio = 3.382 × 0.013062 × (axial length − 1.82), where 3.382 represents the magnification factor as determined by the camera of the OCT imaging system, and the remaining part accounts for the eye-specific magnification factor [24,25]. All OCT images were processed further to enhance image quality and ensure accurate comparisons by aligning volumes to a baseline volume. Specifically, the OCT volume obtained from the MA task was first aligned with the baseline volume through a rigid registration. The registered MA volume was then resampled to ensure each 2D B-scan was at the same location of the baseline volumes. This process ensured that subsequent analysis and comparison occurred between two conditions at the same location. The registration and resampling were conducted using open-source software 3D Slicer version 5.6.1 (Brigham and Women’s Hospital, Boston, MA, USA) [26]. Subsequently, all the images underwent denoising through anisotropic diffusion [27] and were then further refined with adaptive compensation [28].

A deep learning model was developed to segment the OCT images to isolate the retina and choroid. To train and validate the model, 8814 OCT images were manually segmented. A U-shaped convolutional neural network (MGUnet) was employed for deep learning [29]. The MGUnet architecture is a sophisticated neural network designed for image segmentation tasks, characterized by its unique integration of a graph convolutional network within a U-shaped framework. The network’s structure is primarily composed of an encoder that extracts features from input images, a multi-scale global reasoning module that employs graph convolutional networks for enhanced spatial reasoning, and a decoder that reconstructs the segmentation output. The architecture also includes skip connections that facilitate the retention of spatial information throughout the network, allowing MGUnet to effectively process and analyze complex image data. The network used the Adam optimizer with a learning rate of 0.001 and a cross-entropy loss function. The Dice coefficient, ranging from 0 to 1, measured the segmentation accuracy, with values above 0.9 indicating excellent overlap between automated and manual segmentations. The retinal layer was automatically segmented from the internal limiting membrane to the middle of the retinal pigment epithelium (RPE) and the choroidal layer from the middle of RPE to the choroid–scleral interface.

Once the model was developed, all baseline and resampled images were segmented using the deep learning model.

### 2.4. Measurement of Choroidal, Retinal Thickness, and Posterior Eye Curvature

The segmentation images were used to measure the average thicknesses of the retina and choroid in the axial direction. These measurements were specifically taken within circles of 1 mm and 3 mm diameters centered on the lowest point of the fovea. This resulted in values termed as RT_1mm_ and RT_3mm_ for the retina and CT_1mm_ and CT_3mm_ for the choroid. 

**Figure 2:**
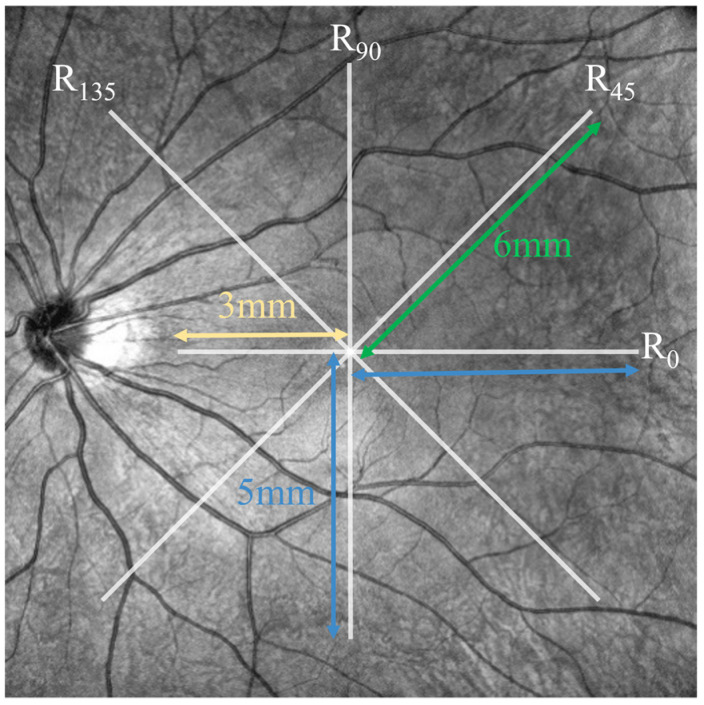
Schematic of the position for calculating best-fitting circles from the upper boundary of the choroid.

To quantify the shape changes in the posterior eye, four radii of the best-fit circles for the upper boundary of the choroid in different directions, which intersect at the lowest point of the macular fovea, were calculated. The horizontal transverse scanning direction of the baseline OCT image was taken as the reference starting direction (i.e., 0°). From the upper boundary of the choroid, the radii of the best-fitting circles R_0_, R_45_, R_90_, R_135_ were determined in the directions perpendicular to the axial direction at 0°, 45°, 90°, and 135°, with counterclockwise angles considered positive. During the fitting process, the radii R_45_, R_90_, R_135_ were determined by all points that were less than 6 mm, 5 mm, and 6 mm in horizontal distance from the lowest point of the fovea, respectively. Meanwhile, R_0_ was determined by the points that were less than 5 mm and 3 mm in horizontal distance from the lowest point of the macular fovea to the temporal and nasal sides, respectively (Figure 2).

### 2.5. Heart Rate Data Analysis

The RR interval data were exported from the Elite HRV application for detailed analysis. For each participant, the RR interval data from the 20 s preceding the end of each OCT acquisition were used. From these data, two key metrics were calculated: the mean HR and the root mean square of the successive differences between adjacent RR intervals (RMSSD). The mean HR provides a measure of the average HR over the specified period. Meanwhile, the RMSSD is a commonly used metric in HR variability studies, estimating the short-term variability in HR and serving as an indicator of cardiac parasympathetic nervous system activity.

### 2.6. Statistical Analysis

The normality of the data distribution was assessed using the Shapiro–Wilk test. The differences in ocular and HR parameters under the MA condition compared to the baseline were assessed with paired t-tests or Wilcoxon signed-rank tests depending on normality of distribution. The correlation between changes in CT and axial length, the correlation between baseline RMSSD and baseline CT, as well as the correlation between changes in CT and changes in HR, were examined using Pearson’s test if both variables followed a normal distribution; otherwise, Spearman’s test was used. Within-session repeatability of the retinal and choroidal thickness measurements was assessed using Bland–Altman analysis, based on the two scans acquired at baseline. Statistical analysis was performed using the SciPy stats package [30]. Data are presented as mean ± standard deviation, unless otherwise specified. *p* < 0.05 was considered statistically significant.

## 3. Results

### 3.1. Subjects and OCT Images

Among the enrolled participants, there were 18 women and 15 men with an average age of 23.546 ± 2.373 years (range, 19 to 29 years). Their average axial length of the left eye was 25.563 ± 1.242 mm.

**Figure 3:**
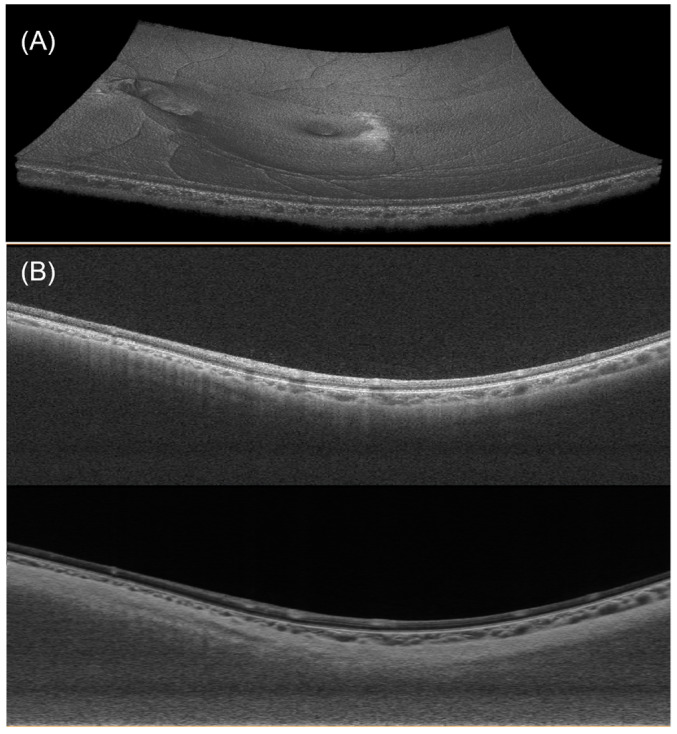
(**A**) Visualization of a 3D OCT dataset. (**B**) A typical B-scan (upper) and its enhanced version after denoising and compensation (lower).

An example of the obtained OCT images is shown in Figure 3A. A typical B-scan and its compensated version are shown in Figure 3B.

### 3.2. Automated Segmentation Model

**Figure 4:**
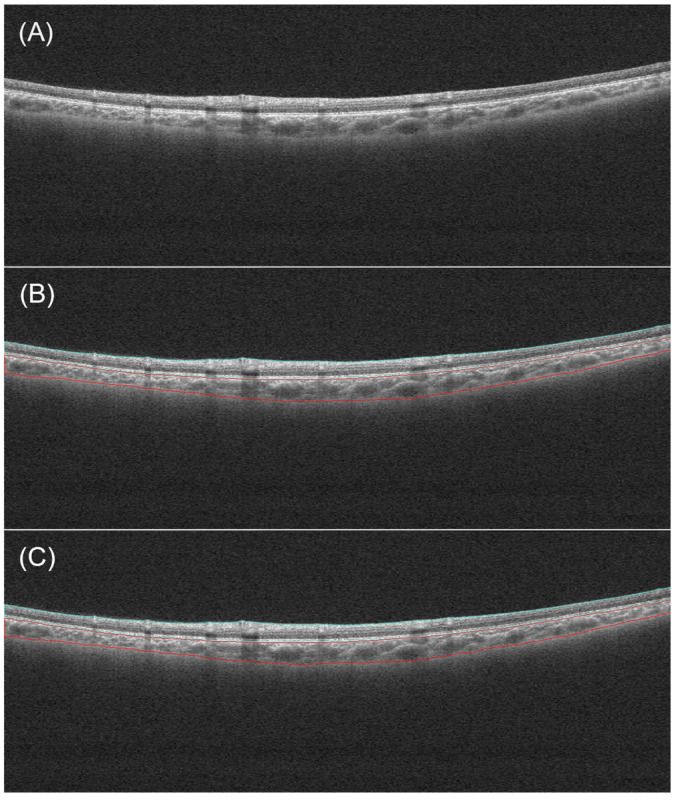
An example of segmentation. (**A**) Original image. (**B**) Manual segmentation result. (**C**) Automatic segmentation result. The blue and red lines respectively indicate the boundaries of the retina and the choroid.

The developed deep learning model achieved a Dice coefficient of 0.969 for retina and 0.965 for choroid in the test dataset. Manual examinations of all automatic segmentations showed that the automated segmentation method accurately segmented the retinal and choroidal layers. Figure 4 shows a comparison of the automated segmentation results and the manual labels.

### 3.3. Within-Session Repeatability

**Figure 5:**
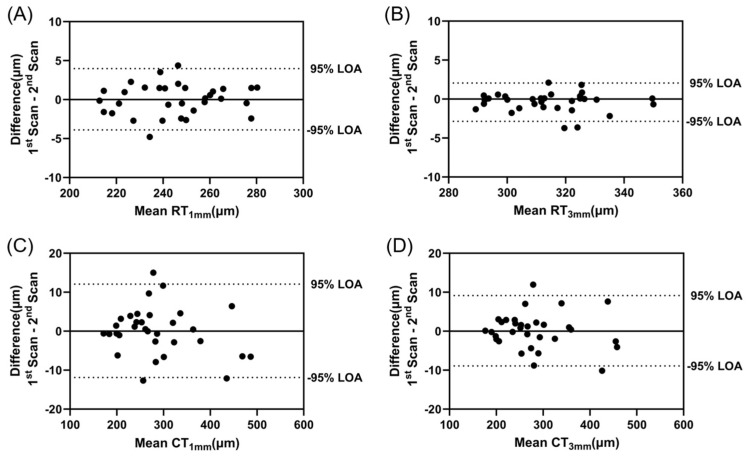
Bland–Altman analysis of the difference between two scans acquired at baseline for (**A**) RT_1mm_, (**B**) RT_3mm_, (**C**) CT_1mm_, and (**D**) CT_3mm_ to assess within-session repeatability. The dotted lines represent 95% limits of agreement.

Bland–Altman analysis was used to assess within-session repeatability of the retinal and choroidal thickness measurements (Figure 5). One scan was excluded from the analysis due to poor image quality. The mean difference between the two baseline scans was 0.046 ± 2.007 µm (95% limits of agreement from −3.887 to 3.979 µm) for RT_1mm_, −0.395 ± 1.253 µm (95% limits of agreement from −2.851 to 2.061 µm) for RT_3mm_, −0.101 ± 6.119 µm (95% limits of agreement from −11.893 to 12.094 µm) for CT_1mm,_ and 0.120 ± 4.604 µm (95% limits of agreement from −8.904 to 9.144 µm) for CT_3mm_.

### 3.4. Changes in Retinal and Choroidal Thickness

The changes in posterior segment ocular parameters observed during the MA task are presented in Table 1. Both CT_1mm_ and CT_3mm_ exhibited significant increases under the MA condition, with average changes of 2.742 ± 7.098 μm (*p* = 0.034) and 3.326 ± 6.143 μm (*p* < 0.001), respectively. No significant changes were observed in the retinal thickness and the radius of the best-fit circle at different positions during the MA task. No significant correlation was observed between axial length and both ΔCT_1mm_ (Pearson *r* = 0.182, *p* = 0.312) and ΔCT_3mm_ (Spearman *r* = 0.092, *p* = 0.609).

### 3.5. Heart Rate Parameters Changes

**Figure 6:**
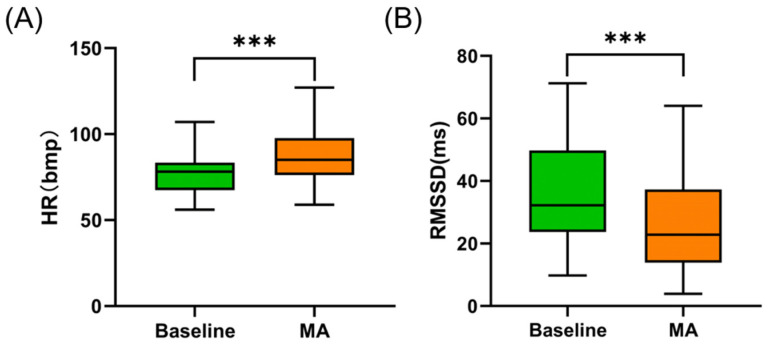
(**A**) Changes in HR under MA. (**B**) Changes in RMSSD under MA. *** indicates *p* < 0.001.

The RR interval data of one participant was excluded due to poor contact. The changes in heart rate parameters under the MA condition are illustrated in Figure 6. Under the MA condition, the HR significantly increased compared to the baseline (from 77.029 ± 12.305 bpm to 86.727 ± 15.833 bpm, *p* < 0.001). The RMSSD decreased from 36.871 ± 17.149 ms to 25.502 ± 13.869 ms during the MA task (*p* < 0.001). No significant correlation was observed between baseline RMSSD and both baseline CT_1mm_ (Spearman *r* = 0.195, *p* = 0.284) and CT_3mm_ (Spearman *r* = 0.192, *p* = 0.291) nor between ΔHR and both ΔCT_1mm_ (Spearman *r* = 0.176, *p* = 0.336) and ΔCT_3mm_ (Spearman *r* = 0.283, *p* = 0.117).

## 4. Discussion

This study provides the first assessment of the choroidal response under acute mental stress. We found that MA induced choroidal thickening, with the stress state confirmed by increased HR and decreased RMSSD.

Previous research has shown differing choroidal response to other stressors. For instance, one study reported a reduction in subfoveal CT in healthy participants after the cold pressor test, a physical stressor involving immersing right hands up to the wrist in cold water for 30 s [31]. The primary reason for the choroidal response differing from this study may be due to the type of stressor, as different stressors require different stress responses [11]. For example, Gregg et al. [32] observed that MA and the cold pressor test elicited different hemodynamic responses in healthy subjects. While both of them increased systolic and diastolic blood pressure, MA led to a rise in HR, stroke volume, and cardiac output without significantly altering total peripheral resistance. In contrast, the cold pressor test caused an elevation in total peripheral resistance with no significant change in the HR, stroke volume, and cardiac output. In addition, several studies have observed that the choroid is thinner during the day with heightened sympathetic nervous system activity and thicker at night when parasympathetic nervous system activity is higher [33,34,35,36,37]. The choroid is one of the most richly vascularized tissues in the human body, with a high blood flow to tissue volume ratio, influenced by systemic hemodynamics [18]. The present study found that choroidal thickening under the MA condition, which is expected to increase sympathetic activity and decrease parasympathetic activity, may be due to the changes in systemic hemodynamics mentioned above [38]. We did not observe a significant correlation between ΔHR and ΔCT, which may be attributed to the inability of a single parameter to adequately represent systemic hemodynamics or the direct influence of the autonomic nervous system on the choroid being a confounding factor.

This study observed that acute mental stress increased CT. While some studies hypothesize that an increase in CT may protect against myopia [39,40], we speculate that the potential influence of long-term or chronic mental stress on the progression of myopia is possible. This is particularly relevant as mental stressors are prevalent in educational settings. The process of adaptation to stressors, referred to as allostasis, involves responses of the autonomic nervous system, hypothalamic–pituitary–adrenal axis, immune system, etc. Yet, adaptation comes at a cost, known as allostatic load. For instance, too much repeated or chronic stress can lead to the wear and tear or imbalance of these systems that promote adaptation, resulting in their failure to shut off or failure to respond [41]. If repeated or chronic mental stress causes systems that participate in adaptation to fail to shut off after activation, it may result in increased sympathetic activity and decreased parasympathetic system activity at rest [13,42,43]. This type of autonomic imbalance may explain some differences between myopia and non-myopia found in past studies. Specifically, numerous studies have found that myopia is associated with a lower tonic accommodation level [44], a thinner choroid [18,45], and reduced ocular blood flow parameters [40,46,47]. Furthermore, results from a study showed that progressive high myopia was linked to lower high-frequency component power (HF) and higher low-frequency component power (LF)/HF ratio in the spectral components of HR variability at baseline status compared to controls [48]. The HF reflects vagal activity (the vagal nerve provides parasympathetic innervation to the heart), and the LF/HF is traditionally considered an indicator of sympathetic–vagal balance [49]. Moreover, a longitudinal study investigated the relationship between the morning urinary catecholamine concentrations—indicators of sympathetic activity—and both CT and axial length elongation in children. They found that the adrenaline concentration was negatively correlated with the CT, and the noradrenaline concentration was positively correlated with both axial length and axial elongation [50]. In addition, repeated or chronic mental stress may also lead to a loss of flexibility in the autonomic nervous system, which is involved in adaptation [51]. Many studies have reported that myopia exhibits a greater level of nearwork-induced transient myopia and a decreased distance accommodative facility compared to emmetropia [52,53,54]. It may be explained by an inflexible parasympathetic withdrawal or sympathetic activation. A study found reduced reactivity of the autonomic nervous system to functional tests in progressive high myopia [48], which may be attributed to a lack of flexibility in adjusting autonomic responses to meet demands.

Here is a hypothesis regarding the potential association between the imbalance of the autonomic nervous system and myopia: The ophthalmic artery and its branches within the choroid are expected to constrict in response to increased sympathetic nervous activity and decreased parasympathetic nervous activity, potentially reducing the blood flow [16,38,55,56]. The potential reduction in blood flow at rest could lead to axial elongation of the eye (i.e., worsening of myopia) through the scleral hypoxia [57]. It is noteworthy that the choroidal blood circulation accounts for roughly 85% of the overall blood flow in the eye, and there is an absence of effective blood flow autoregulation [38,56]. A study found no abnormal serum levels of certain stress mediators, including cortisol, Adrenocorticotropic Hormone, growth hormone, and prolactin, in myopia [58]. This may be attributed to the fact that levels of these stress mediators do not reflect sympathetic nervous system activity as well as catecholamines.

It is worth mentioning that, in addition to repeated mental stress, many events in daily life that elevate the activities of physiological systems are also considered to contribute to allostatic load [59]. Whether long-term ocular accommodation contributes to an increase in allostatic load remains to be further investigated. Yet, a study found that with increased ocular accommodative demand, there was a decrease in HF of the HR variability spectrum and an increase in HR [60].

If the stress or the imbalance in the autonomic nervous system caused by stress is contributing to myopia, certain pathways that prevent them may potentially help in myopia control. Several strategies are considered to reduce the harmful effects of work stress: physical exercise, meditation, and consuming omega-3 fatty acids and vitamin D [61]. Additionally, some technologies have been employed to modify cardiac autonomic balance, such as music embedded with binaural beat technology, bidirectional sensory motor rhythm training, heart rate variability biofeedback, and transcutaneous vagal nerve stimulation [62].

This study presents several limitations. Firstly, the subjects recruited have reached an age where refractive states are relatively stable. Future studies may consider replicating these experiments in school-aged children. Secondly, the time span for observing the impact of mental stress on the choroid was not long enough. Future research should consider observing the cumulative effects of repeated mental stress on the choroid or long-term monitoring of stress events experienced by school-aged children to evaluate their relationship with the progression of myopia. Thirdly, the orientation of images was not corrected for individual anatomical differences based on the relative positions of the fovea and optic disc, which may influence the assessment of changes in the radius of curvature. Future studies could correct images by identifying the relative positions of the Bruch’s membrane opening and the fovea. Fourthly, the present study was primarily focused on the macular choroidal thickness. Future research may consider further subdivision of the thickness regions and investigate a broader range of thickness to better understand the effects of mental stress on the choroid. Fifthly, the refractive state of the subjects was not assessed. Future research should consider the refractive status. Sixthly, this study only measured a limited number of heart rate parameters. Future research could measure a broader range of systemic hemodynamic parameters and more autonomic markers to better understand the primary factors of choroidal response to mental stress.

In conclusion, we found that short-term mental stress (<2 min) caused an increase in CT. An increase in HR and a decrease in RMSSD were observed under mental stress, with no correlation found between ΔCT and ΔHR. While some studies propose that a thicker choroid is associated with slower myopia progression, we speculate that long-term or chronic mental stress may potentially affect myopia progression. It may lead to an imbalance in the autonomic nervous system, potentially affecting the choroidal blood flow and thus influencing the progression of myopia.

## Figures and Tables

**Table 1:** Changes in posterior segment ocular parameters under MA (*n* = 33).

Parameter	Baseline	MA	Difference	*p*
RT_1mm_ (μm)	245.717 ± 19.420	245.949 ± 19.613	0.232 ± 2.352	0.575 ^a^
RT_3mm_ (μm)	314.906 ± 15.821	314.894 ± 16.007	−0.012 ± 1.510	0.963 ^a^
CT_1mm_ (μm)	288.091 ± 82.384	290.833 ± 81.024	2.742 ± 7.098	0.034 ^a^*
CT_3mm_ (μm)	282.954 ± 76.694	286.280 ± 75.912	3.326 ± 6.143	<0.001 ^b^*
R_0_ (mm)	17.746 ± 4.988	17.625 ± 4.958	−0.122 ± 0.791	0.537 ^b^
R_45_ (mm)	22.074 ± 6.855	21.976 ± 7.390	−0.098 ± 1.573	0.221 ^b^
R_90_ (mm)	22.739 ± 9.699	22.698 ± 10.008	−0.040 ± 1.145	0.822 ^b^
R_135_ (mm)	19.398 ± 5.539	19.253 ± 5.113	−0.145 ± 0.849	0.888 ^b^

Abbreviations: RT, retinal thickness; CT, choroidal thickness; R, radius of the best-fit circle. Variables are expressed as mean ± standard deviation. * indicates *p* < 0.05. ^a^ Paired t-test, ^b^ Wilcoxon signed-rank test.

## Data Availability

The original contributions presented in the study are included in the article, further inquiries can be directed to the corresponding author.
